# Effects of a Cognitive Bias Modification Training on Resting State EEG Microstates in Patients with MDD and Healthy Controls

**DOI:** 10.1192/j.eurpsy.2024.1116

**Published:** 2024-08-27

**Authors:** J. Kesik, Z. Kratochwil, D. Keskin-Gökcelli, B. Müller, T. Frodl

**Affiliations:** ^1^Department of Psychiatry, Psychotherapy and Psychosomatics, RWTH Aachen University, Aachen; ^2^Department of Psychiatry and Psychotherapy, LVR-University Hospital Essen, Duisburg-Essen , Germany

## Abstract

**Introduction:**

Major Depressive Disorder (MDD) is associated with a high burden of disease and notable economic costs. Standard treatments (e.g. medication or cognitive therapy) have been shown to be effective, but some patients remain unresponsive. With the knowledge that MDD patients have been shown to display an attentional cognitive bias towards negative stimuli, Cognitive Bias Modification (CBM)-training to focus attention on positive information is thought to improve emotional processing and depressive symptoms. Some studies imply reduced duration and occurrence of microstate D in MDD compared to healthy controls. However, the effect of CBM on microstates is still unclear.

**Objectives:**

(1) To replicate previous findings that duration and occurrence of microstate D is reduced in patients with MDD compared to healthy controls in an independent sample and (2) to investigate the effect of an active CBM-training versus a control-training on microstates and its association with symptom improvements.

**Methods:**

Thirty patients receiving outpatient treatment with MDD according to DSM V (aged 18-60) will be recruited in Essen and Aachen. The control group will consist of 30 healthy age-and-sex-matched participants. Psychological testing will be administered and all participants will be randomized to either an active or a control training. During the next visit, resting state EEG and a GoNoGo Task with positive, neutral and negative pictures will be measured. The participants will take a tablet home to undergo 10 sessions of CBM within 14 days. The training will be consisted of a dot-probe-task. In the active condition the probe will be more likely to appear behind a positive versus a neutral picture, while appearing randomly in the control condition. After 14 days, a second EEG will be recorded.

**Results:**

Differences in duration and occurrence of microstate D between patients and healthy controls will be analyzed by conducting ANCOVAs with age and sex as covariates. ANCOVAs for repeated measurements will be calculated to study effects of time (pre- vs. post-training) and group (patients vs. healthy controls in active training; patients in active vs. patients in control-training), on duration and occurrence of microstate D.

**Conclusions:**

CBM-training is proposed to be an effective treatment option for MDD patients, reflected in a reduced topographical bias of microstate D in EEG.

**Disclosure of Interest:**

None Declared

